# Salt-Tolerant Non-Crosslinked Polymer Networks for High-Temperature Weighted Fracturing Fluids in Deep Hydrocarbon Reservoirs

**DOI:** 10.3390/gels12100905

**Published:** 2026-10-08

**Authors:** Jv Liu, Xin Qiao, Jianxin Peng, Lili Yan, Qingsong Cheng, Fei Peng, Bowen Zhong, Shulong Chen, Lingfeng Wu, Yang Bai

**Affiliations:** 1 CNPC Tarim Oilfield Company, Korla 841000, China; liuju8-tlm@petrochina.com.cn (J.L.); qiaoxin-tlm@petrochina.com.cn (X.Q.); pengjx-tlm@petrochina.com.cn (J.P.); lilili-tlm@petrochina.com.cn (L.Y.); chqs-tlm@petrochina.com.cn (Q.C.); pengf-tlm@petrochina.com.cn (F.P.); zhongbw1-tlm@petrochina.com.cn (B.Z.); chensl3-tlm@petrochina.com.cn (S.C.); 2 R&D Center for Ultra Deep Complex Reservoir Exploration and Development, CNPC, Korla 841000, China; 3 Engineering Research Center for Ultra-Deep Complex Reservoir Exploration and Development, Xinjiang Uygur Autonomous Region, Korla 841000, China; 4 Xinjiang Key Laboratory of Ultra-Deep Oil and Gas, Korla 841000, China; 5 School of Oil & Natural Gas Engineering, Southwest Petroleum University, Chengdu 610500, China; baiyanghyq@foxmail.com

**Keywords:** weighted fracturing fluid, non-crosslinked dynamic network, hydrophobically associating polymer, AMPS, potassium formate, drag reduction, proppant suspension

## Abstract

High-density potassium formate fracturing fluids require polymers that hydrate in concentrated brine while providing low friction and proppant suspension at high temperature. We hypothesized that the 2-acrylamido-2-methylpropanesulfonic acid (AMPS) content balances salt-tolerant hydration against molar-mass-dependent chain entanglement, enabling a reversible non-crosslinked network. Five hydrophobically associating copolymers based on acrylamide (AM) and AMPS, with AMPS feed contents of 5.5–23.8 wt%, were synthesized by low-temperature micellar copolymerization followed by post-hydrolysis. Polymer structure, molar mass, and solution conformation were determined by proton nuclear magnetic resonance spectroscopy and size-exclusion chromatography coupled with multi-angle light scattering. Hydration and thickening were evaluated in 1.40 g/cm^3^ potassium formate brine, followed by rheological testing at 160 °C, pipe-flow drag-reduction testing, and static proppant-suspension testing. Increasing the AMPS feed content decreased the weight-average molar mass from 25.83 × 10^6^ to 9.26 × 10^6^ g/mol, increased the chain-conformation exponent from 0.570 to 0.610, and shortened the time required to reach 90% hydration (t_90_) from approximately 118 to 48 min. SN-4, containing 17.9 wt% AMPS, provided the best overall balance. A 0.60 wt% SN-4 fluid retained approximately 63 mPa·s after shearing at 160 °C and 170 s^−1^ and recovered 92.1% of its viscosity after 300 s. Fresh and thermally sheared–recovered fluids maintained drag-reduction rates above 60%, and the recovered fluid reduced proppant settling velocity by 97.4%. These results support the proposed balance and demonstrate the potential of this reversible network for high-temperature weighted fracturing fluids in deep hydrocarbon reservoirs.

## 1. Introduction

As oil and gas exploration and development advance toward deep, ultradeep, and geologically complex reservoirs, the high formation and treating pressures associated with increasing burial depth impose more demanding performance requirements on fracturing-fluid systems [1]. Conventional low-density fracturing fluids rely mainly on surface pumping pressure to overcome frictional losses along the wellbore and the reservoir breakdown pressure. In deep and ultradeep wells, this can result in high wellhead treating pressures, heavy equipment loads, and a restricted safe operating window [2,3]. Introducing a high-density soluble salt into a fracturing fluid increases fluid density and allows hydrostatic head to offset part of the required bottomhole pressure, thereby reducing surface treating pressure and improving operating conditions in ultradeep-well fracturing [4,5,6,7]. Consequently, weighted fracturing fluids that combine high density, favorable rheological properties, and low friction have become an important research direction for stimulation of deep hydrocarbon reservoirs [8].

Compared with conventional water-based fracturing fluids, weighted fracturing fluids usually contain high concentrations of inorganic or organic salts, and their high ionic strength substantially changes polymer hydration and chain conformation in solution. In concentrated brine, abundant ions compete with hydrophilic polymer groups for water molecules, weaken the polymer hydration shell, and cause electrostatic screening of charged groups [9,10]. The chains consequently contract, their hydrodynamic volume decreases, dissolution slows, thickening capacity declines, and viscosity retention at high temperature becomes inadequate [11,12,13]. These effects are especially pronounced in non-crosslinked fracturing fluids whose structure depends on polymer-chain expansion and intermolecular association. Maintaining polymer solubility, thickening capacity, temperature and shear resistance, and drag-reduction performance simultaneously in a high-salinity weighted medium is therefore a central challenge in the design of non-crosslinked weighted fracturing fluids [7,13].

Polyacrylamide and its derivatives are widely used as water-soluble thickeners and drag reducers in fracturing fluids because of their high molar mass and good water solubility [13,14]. However, acrylamide-dominant polymers readily undergo chain coiling, hydrolysis, and thermal degradation under high-temperature, high-salinity conditions, and their rheological performance is often insufficient for such complex environments [11,12,13]. 2-Acrylamido-2-methylpropanesulfonic acid (AMPS) contains a bulky sulfonic acid group and exhibits strong hydration as well as good salt and temperature tolerance [15,16,17,18]. Incorporating AMPS into a polyacrylamide chain is therefore a common strategy for improving polymer stability at high temperature and salinity. AMPS units strengthen interactions between the polymer chain and water molecules and partly mitigate the adverse effect of concentrated electrolytes on chain conformation [15,16,17,18].

Nevertheless, increasing the AMPS content does not necessarily produce a continuous improvement in overall polymer performance. During polymerization, differences in monomer reactivity and in chain-transfer and termination behavior affect the final molar mass and molecular architecture [13,15]. For a drag-reducing thickener, high molar mass increases chain hydrodynamic dimensions and solution viscosity and enables stretched macromolecular chains to suppress turbulent fluctuations, thereby improving drag reduction [19,20,21,22]. An excessive functional-monomer content may therefore improve salt tolerance while lowering polymer molar mass, creating a new conflict among salt tolerance, thickening, and drag reduction [13,15]. Rational control of the AMPS content, and thus a balance between molar mass and functional-group content, is essential when designing drag-reducing thickeners for concentrated brines.

In addition, introducing a small fraction of hydrophobic groups into the polymer backbone enables reversible hydrophobic association and formation of a dynamic physical network, increasing structural strength and viscosity recovery [23,24,25,26]. Unlike conventional chemically crosslinked systems, hydrophobically associating polymers form networks predominantly through reversible intermolecular physical interactions [23,24,25]. Associative structures dissociate under shear and reform as shear weakens, producing a favorable shear-responsive character [26,27]. The synergy between macromolecular-chain expansion and the hydrophobic-association network may also allow these polymers to function as drag reducers while retaining thickening capacity [14,26,27]. In weighted fracturing fluids, the combined design of salt-tolerant groups, an ultrahigh-molar-mass backbone, and an appropriate fraction of hydrophobic associating groups could therefore improve both thickening and drag reduction in concentrated brine [15,16,18,26].

On this basis, acrylamide (AM), AMPS, and sodium N-hexadecyl-2-acrylamidoethanesulfonate were used as the principal functional monomers to prepare a hydrophobically associating drag-reducing thickener for weighted fracturing fluids by low-temperature-initiated micellar copolymerization followed by post-hydrolysis. A series of model polymers with different salt-tolerant-group contents and molar masses was obtained by varying the AMPS feed content, enabling systematic evaluation of the relationship between AMPS content and polymer molar mass. The synthesized polymers were characterized by proton nuclear magnetic resonance spectroscopy (^1^H NMR) to qualitatively identify characteristic polymer-backbone and AMPS-related proton signals, and by size-exclusion chromatography coupled with multi-angle light scattering (SEC–MALS) to determine their molar-mass distributions and solution conformations. Their hydration, thickening, drag-reduction, proppant-suspension, and thermal and salt-resistance properties were subsequently evaluated under weighted-fracturing-fluid conditions. The objective was to clarify how variations in AMPS feed content, together with the associated changes in molar mass and chain conformation, influence macroscopic performance, thereby providing a basis for designing non-crosslinked polymers for high-temperature, high-salinity weighted media.

## 2. Results and Discussion

### 2.1. Molecular Design and Composition of the Model Polymers

To clarify how the content of salt-tolerant sulfonate units affects polymer molecular structure and performance in concentrated brine, a series of model polymers was designed and prepared with the AMPS feed content as the principal variable, while the hydrophobic-monomer feed, target degree of hydrolysis, and other synthesis conditions were kept essentially constant. The polymers were synthesized by low-temperature-initiated micellar copolymerization followed by post-hydrolysis, as described in Section 4.2.1.

Within this molecular design, AM units form the water-soluble polymer backbone and provide the chain dimensions and hydrodynamic volume required for thickening. AMPS side groups contain strongly hydrated sulfonate groups and a sterically bulky gem-dimethyl structure, which favor hydration and conformational stability in concentrated brine [15,16,17,18]. Sodium acrylate units formed during post-hydrolysis further increase polymer hydrophilicity and segmental charge density. The long alkyl chain of sodium N-hexadecyl-2-acrylamidoethanesulfonate provides association sites for formation of an intermolecular dynamic physical network through reversible hydrophobic association [28,29,30,31]. Thus, the model polymers combine a water-soluble backbone, salt-tolerant sulfonate groups, hydrophilic carboxylate groups, and long-chain hydrophobic associating groups.

The monomer compositions and basic structural parameters of the model polymers are summarized in Table 1. The monomer compositions, degrees of hydrolysis, and weight-average molar masses (M_w_) of the model polymers are summarized in Table 1. With the hydrophobic-monomer feed fixed at 0.4% and the target degree of hydrolysis at 30%, AMPS feed contents of 5.5%, 9.0%, 13.0%, 17.9%, and 23.8% yielded SN-1, SN-2, SN-3, SN-4, and SN-5, respectively; the AM feed content was reduced correspondingly as the AMPS content increased. The measured degrees of hydrolysis were 29.1–31.2%, close to the target value, showing that the extent of post-hydrolysis was similar across the series. The series therefore primarily represents a gradient in the AMPS-to-AM feed ratio and is suitable for examining the effects of AMPS feed content on molecular structure and performance in concentrated brine.

**Figure 1:**
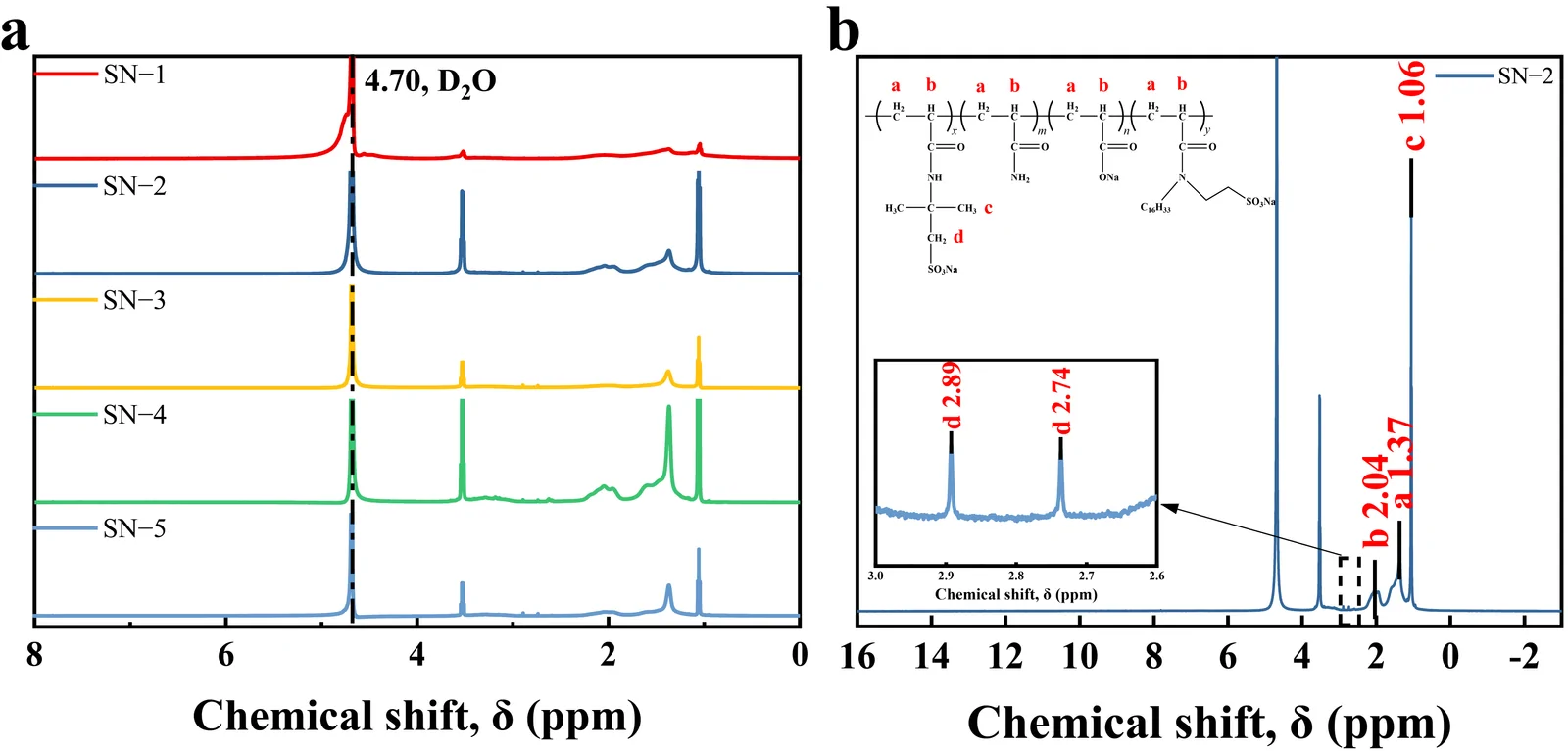
^1^H NMR spectra and structural assignments of model polymers with different AMPS feed contents. (**a**) ^1^H NMR spectra of SN−1, SN−2, SN−3, SN−4, and SN-5; (**b**) ^1^H NMR spectrum and structural assignment of representative sample SN−2, with the designed polymer structure and proton labels in the upper-left inset and an enlarged view of the δ 2.6−3.0 ppm region in the lower-left inset. The lowercase letters a−d denote the assigned proton environments: a and b correspond to the polymer-backbone methylene and methine protons, respectively; c corresponds to the methyl protons in the AMPS units; and d corresponds to the methylene protons adjacent to the sulfonate group in the AMPS units.

The ^1^H NMR spectra of the model polymers are shown in Figure 1a. The principal characteristic signals occur at essentially the same positions for all samples, indicating a common basic copolymer structure. To assign the proton signals more clearly, Figure 1b correlates the ^1^H NMR spectrum of representative sample SN-2 with its designed structure. The upper-left inset shows the designed polymer structure and proton labels, the main panel shows the full spectrum of SN-2, and the lower-left inset enlarges the δ 3.0–2.6 ppm region. The intense peak near δ 4.70 ppm is assigned to residual partially deuterated water (HDO). The signal at δ 2.04 ppm is assigned to backbone methine proton b, the broad peak at δ 1.37 ppm to backbone methylene protons a, and the signal at δ 1.06 ppm to the gem-dimethyl protons c of the AMPS side group. In the enlarged region, the signals at δ 2.89 and 2.74 ppm are both assigned to methylene protons d adjacent to the sulfonate group in the AMPS side chain. These characteristic a–d signals agree with the designed structure and confirm incorporation of AMPS units into the polymer chains. Moreover, no pronounced residual vinyl-proton signals were observed between δ 5.0 and 7.0 ppm, indicating that the polymerizable double bonds of the monomers were essentially consumed.

The ^1^H NMR spectra in this study were used primarily for qualitative confirmation of the basic chemical structures and characteristic proton signals of the model polymers. Because reliable quantitative integration was not performed under uniform acquisition conditions, neither peak heights nor integrated areas were used to calculate the actual AMPS content of the polymers; all AMPS contents reported herein refer to monomer feed contents. Taken together, the formulation design and ^1^H NMR results show that the model polymers share the same basic chemical-structure type while differing in AMPS feed composition, providing an appropriate sample set for evaluating how the AMPS feed content affects molar mass, chain structure, hydration, dissolution, and thickening in concentrated brine.

### 2.2. Effect of AMPS Content on Polymer Molar Mass and Chain Structure

**Figure 2:**
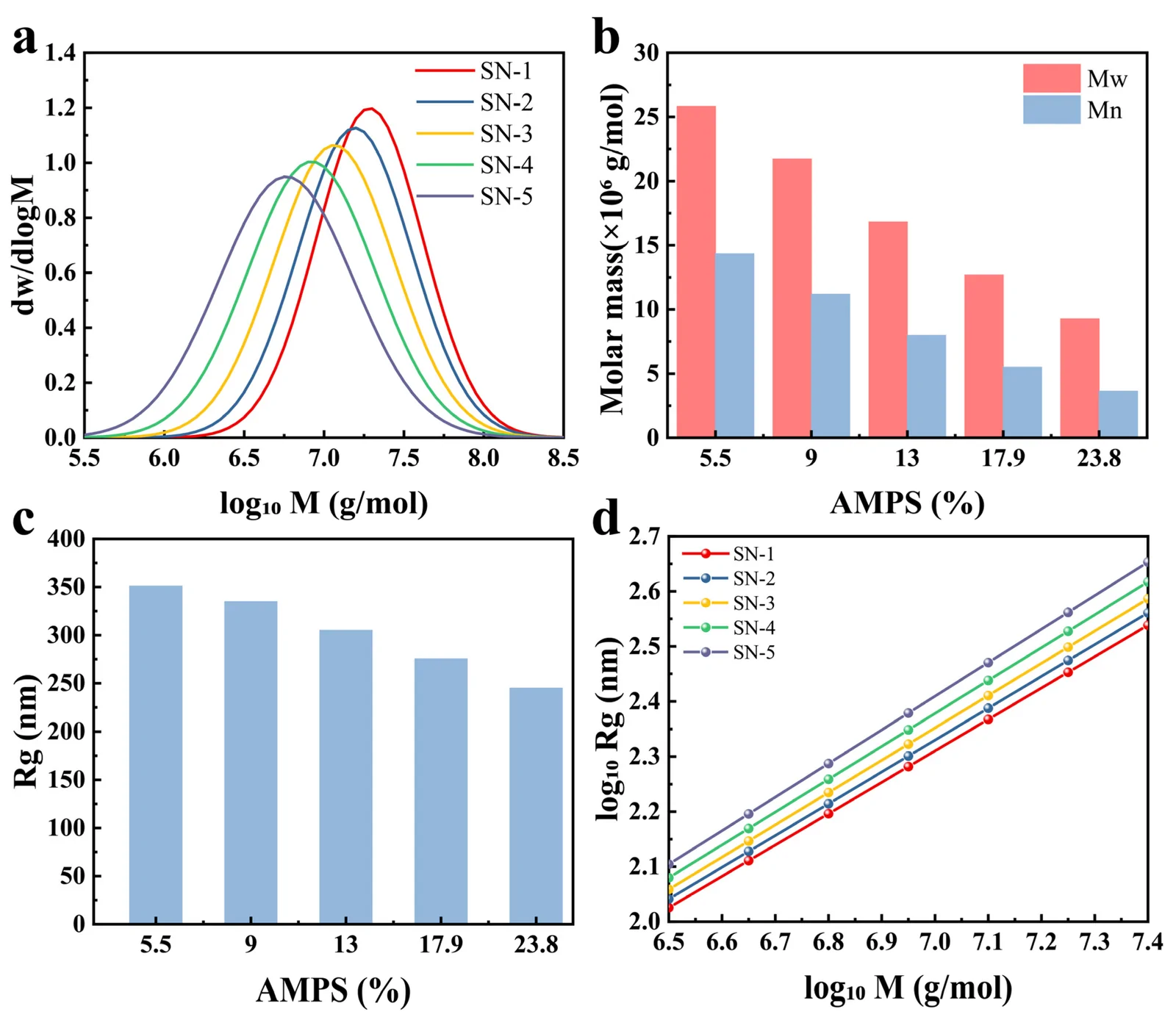
Effects of AMPS content on polymer molar mass and chain conformation. (**a**) Area-normalized molar mass distributions; (**b**) variation in M_w_ and M_n_ with AMPS feed content; (**c**) variation in mean radius of gyration, R_g_, with AMPS feed content; and (**d**) conformational relationship between log_10_R_g_ and log_10_M.

The five model polymers were characterized by size-exclusion chromatography coupled with multi-angle light scattering (SEC–MALS) to determine their weight-average molar mass (M_w_)), number-average molar mass (M_n_), dispersity ($Đ=(M_{w}/M_{n})$), and radius of gyration (R_g_), and to examine their molar-mass distributions and solution conformations (Figure 2). As shown in Figure 2a, all five polymers exhibited continuous, unimodal molar mass distributions, with no distinct low-molar-mass fraction or anomalously high-molar-mass aggregate peak. As the AMPS feed content increased from 5.5% to 23.8%, the distribution peaks shifted progressively toward lower molar mass, with the peak log_10_M decreasing from approximately 7.28 to 6.76, while the distributions became broader. Thus, increasing the AMPS content shortened the polymer chains overall and increased heterogeneity among chain-length fractions.

Figure 2b further shows the variation in M_w_ and M_n_ with AMPS feed content. At AMPS contents of 5.5%, 9.0%, 13.0%, 17.9%, and 23.8%, M_w_ values were 25.83 × 10^6^, 21.73 × 10^6^, 16.82 × 10^6^, 12.69 × 10^6^, and 9.26 × 10^6^ g/mol, respectively, whereas M_n_ values were 14.35 × 10^6^, 11.18 × 10^6^, 7.98 × 10^6^, 5.50 × 10^6^, and 3.63 × 10^6^ g/mol. Relative to SN-1, M_w_ and M_n_ of SN-5 decreased by 64.1% and 74.7%, respectively, indicating that M_n_ was more sensitive to AMPS content. Accordingly, the dispersity, M_w_/M_n_, increased from 1.80 to 2.55, consistent with the progressive broadening in Figure 2a. With the other polymerization conditions held constant, increasing the AMPS fraction may alter the relative rates of chain propagation, transfer, and termination in the copolymerization system, thereby shortening the effective propagation length and increasing heterogeneity in the resulting molar mass distribution [15,18,28].

The change in overall polymer dimensions is shown in Figure 2c. As the AMPS content increased, the mean R_g_ decreased from 350.97 nm for SN-1 to 334.70, 304.98, 275.46, and 244.94 nm for SN-2 through SN-5, respectively, representing an overall decrease of 30.2%. This trend follows that of M_w_, indicating that lower molar mass was the principal cause of the decrease in overall coil size. However, because R_g_ depends on both molar mass and chain conformation, a decrease in mean R_g_ cannot be interpreted directly as chain contraction [32,33]. Notably, the reduction in R_g_ was substantially smaller than that in M_w_, suggesting that AMPS-induced chain expansion partly offset the effect of shorter chain length on coil dimensions.

To decouple the effect of molar mass, Figure 2d plots log_10_R_g_ against log_10_M over a common molar mass range. All five polymers showed an approximately linear relationship consistent with the scaling law R_g_ = KM^ν^ [32,33]. The conformation exponents ν for SN-1 through SN-5 were 0.570, 0.578, 0.586, 0.597, and 0.610, respectively, and at a given molar mass R_g_ generally followed SN-5 > SN-4 > SN-3 > SN-2 > SN-1. These ν values indicate predominantly flexible-coil conformations in the mobile phase used, whereas the increase in ν with AMPS content indicates progressively greater chain expansion, possibly because of stronger hydration and intrachain electrostatic repulsion at higher sulfonate-group density [15,33]. Increasing the AMPS content therefore produced two competing effects: it lowered polymer molar mass and reduced overall coil dimensions, but increased chain expansion at a given molar mass. Mean R_g_ alone is consequently insufficient for assessing chain conformation; the molar-mass–size scaling relationship must also be considered.

### 2.3. Effect of AMPS Content on Polymer Hydration and Dissolution in Concentrated Brine

**Figure 3:**
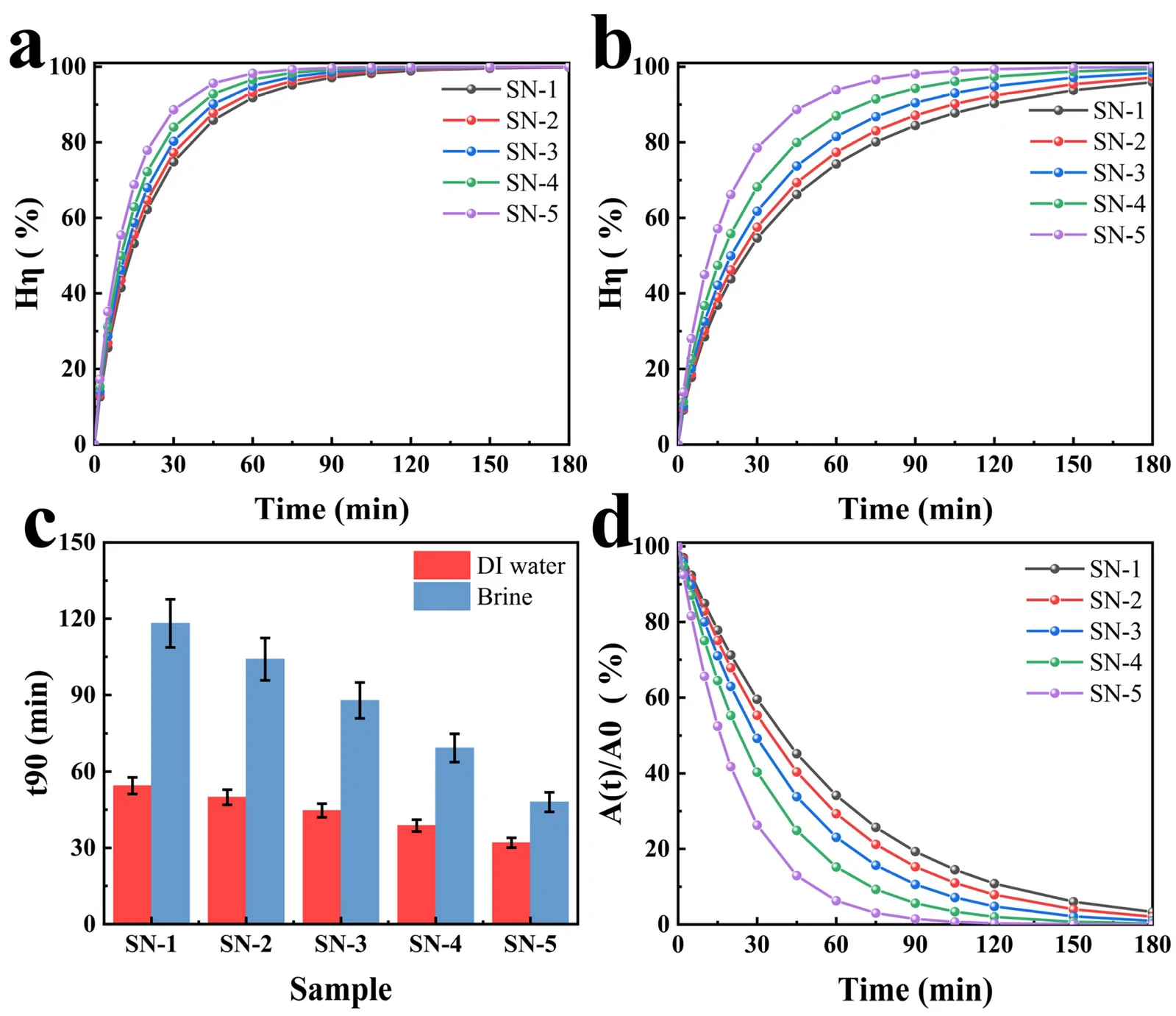
Effects of AMPS content on polymer hydration and dissolution. (**a**) Normalized viscosity development in deionized water; (**b**) normalized viscosity development in 1.40 g/cm^3^ potassium formate brine; (**c**) t_90_ in deionized water and potassium formate brine, with error bars representing standard deviations; and (**d**) residual undissolved-particle area fraction as a function of time in potassium formate brine.

To evaluate how AMPS content affects hydration and dissolution, the viscosity-based degree of hydration, $H_{\eta }\left(t\right)$, and the time required to reach 90% hydration, $t_{90}$, were determined in deionized water and 1.40 g/cm^3^ potassium formate brine. The dissolution state was assessed using the residual undissolved-particle area fraction, $A\left(t\right)/A_{0}$ (Figure 3). As shown in Figure 3a, the normalized viscosity, H_η_, of all five polymers increased rapidly with time in deionized water and gradually approached a plateau. As the AMPS content increased, the hydration curves shifted progressively to the left, and the hydration rate followed SN-5 > SN-4 > SN-3 > SN-2 > SN-1. Thus, higher AMPS contents accelerated viscosity development.

Compared with those in deionized water, the H_η_ curves of all samples shifted markedly to the right in potassium formate brine, showing that the concentrated brine delayed water uptake, particle swelling, and chain release (Figure 3b). After 60 min of hydration, H_η_ values for SN-1 through SN-5 were approximately 74%, 78%, 82%, 87%, and 94%, respectively; thus, samples with higher AMPS contents retained relatively rapid hydration in brine. After 180 min, H_η_ exceeded 95% for all samples, indicating that, under the present conditions, concentrated brine mainly slowed approach to the hydrated state rather than preventing eventual hydration.

Figure 3c compares t_90_ in deionized water and potassium formate brine. The t_90_ values for SN-1 through SN-5 were approximately 54, 50, 45, 39, and 32 min in deionized water and increased to approximately 118, 104, 88, 69, and 48 min in brine. Relative to deionized water, the increases in t_90_ were approximately 64, 54, 43, 30, and 16 min, respectively, and the brine-to-water t_90_ ratio decreased from approximately 2.17 for SN-1 to approximately 1.50 for SN-5. The inhibitory effect of brine on hydration therefore weakened as the AMPS content increased. Raising the AMPS content from 5.5% to 23.8% reduced t_90_ by approximately 40.7% in deionized water and by approximately 59.3% in brine, demonstrating that the benefit of AMPS was more pronounced in the high-salinity medium. Error bars represent the standard deviation of replicate t_90_ measurements; their ranges did not alter the overall ranking of the samples.

The residual undissolved-particle area results agreed well with the normalized viscosity and t_90_ data. As shown in Figure 3d, A(t)/A_0_ decreased continuously with time for all samples, with the same rate order of SN-5 > SN-4 > SN-3 > SN-2 > SN-1. At 60 min, the residual areas for SN-1 through SN-5 were approximately 35%, 30%, 23%, 15%, and 6%, respectively; after 120 min, they had decreased to approximately 11%, 8%, 5%, 2%, and less than 1%. SN-5 exhibited the fastest particle breakup and disappearance, whereas substantial undissolved particles remained for SN-1 over the same period. Thus, increasing the AMPS content accelerated not only macroscopic viscosity development but also water penetration into the particles, particle disintegration, and polymer-chain diffusion into solution.

The changes in hydration rate in concentrated brine likely reflect both the strong hydration of sulfonate groups and the concurrent change in polymer molar mass. Salt ions compress the diffuse electrical double layer around polymer chains and compete for free water, weakening hydration of carboxylate and amide groups and slowing water diffusion into the particles [9,10,33]. The sulfonate group of AMPS remains strongly dissociated and hydrated, while its bulky side group helps resist salt-induced chain contraction [15,16,17,18]. Increasing the AMPS content therefore improved polymer hydration in concentrated brine and reduced the associated delay.

The results in Section 2.2 also show that the increase in AMPS content was accompanied by a decrease in polymer molar mass. Using the values consistently reported in Table 1, the molar mass decreased from 25.83 × 10^6^ to 9.26 × 10^6^ g/mol. Lower molar mass reduces interchain entanglement and segmental diffusion resistance and can therefore shorten hydration and dissolution times [34]. The improved hydration of this sample series should consequently be understood as the combined effect of increased AMPS content and decreased molar mass rather than being attributed to a single structural factor. In addition, H_η_ is normalized to the plateau viscosity of each sample; it primarily describes hydration rate and does not directly compare the final absolute viscosities of different samples.

### 2.4. Effect of AMPS Content on Thickening and Salt-Tolerant Stability

**Figure 4:**
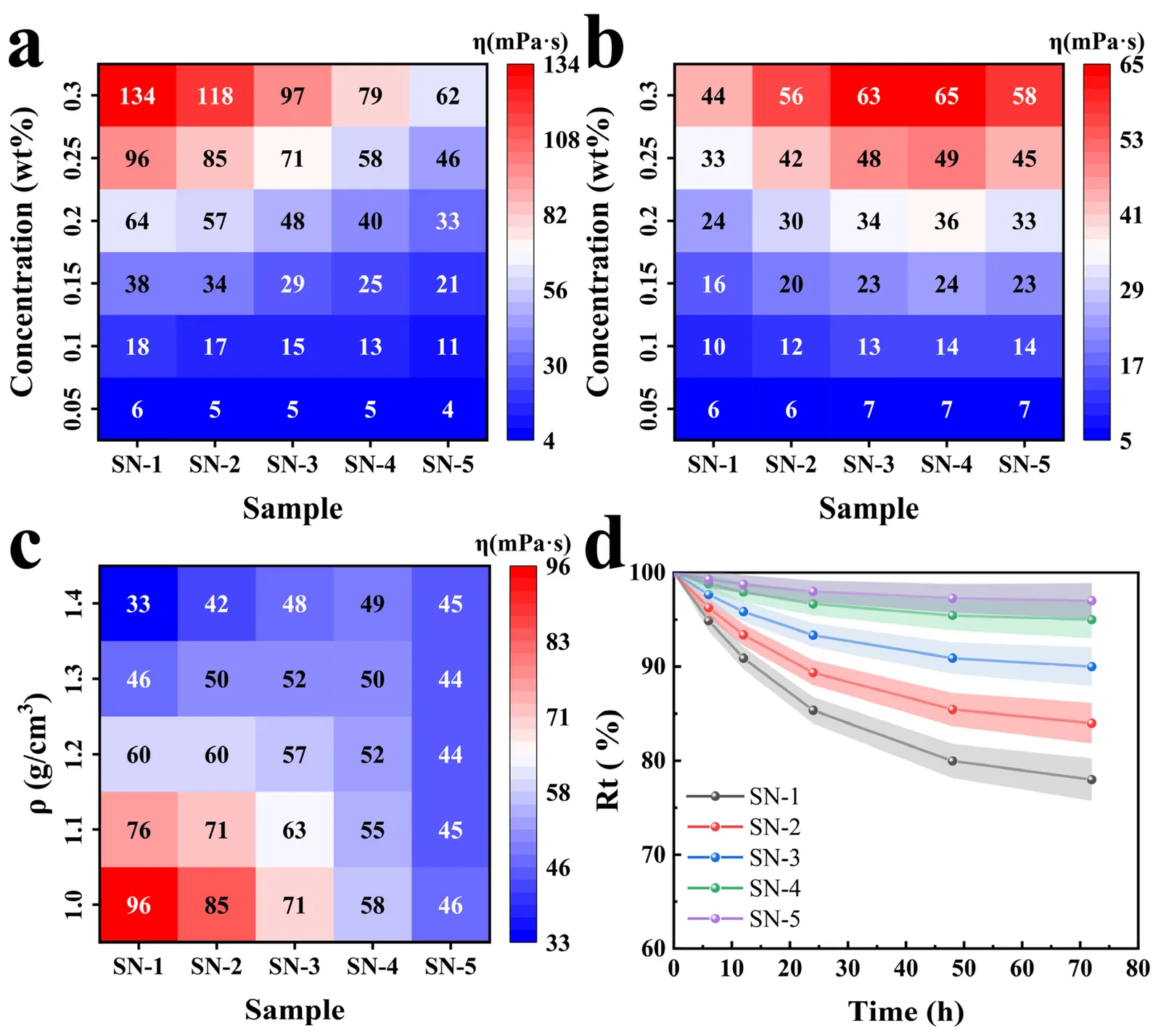
Effects of AMPS content on polymer thickening and salt-tolerant stability. (**a**) Apparent viscosity of polymer solutions of different concentrations in deionized water; (**b**) apparent viscosity of polymer solutions of different concentrations in 1.40 g/cm^3^ potassium formate brine; (**c**) apparent viscosity of 0.25 wt% polymer solutions in potassium formate brines of different densities; and (**d**) viscosity retention during storage in 1.40 g/cm^3^ potassium formate brine. The values in panels (**a**–**c**) are the means of three independent measurements. In panel (**d**), solid lines and symbols represent the mean values, and the shaded regions indicate ±1 SD (*n* = 3).

Figure 4 compares the thickening performance, brine-density sensitivity, and storage stability of the polymers in deionized water and potassium formate weighted brine. As the AMPS feed content increased from 5.5 to 23.8 wt%, the samples progressed from SN-1 to SN-5. As shown in Figure 4a, the apparent viscosity of every polymer in deionized water increased substantially over the concentration range of 0.05–0.30 wt%, with a steeper increase at higher concentrations, indicating stronger interchain entanglement and intermolecular interactions [15,18,23]. At a given concentration, thickening capacity generally followed SN-1 > SN-2 > SN-3 > SN-4 > SN-5. At 0.30 wt%, for example, the apparent viscosities were 134, 118, 97, 79, and 62 mPa·s, respectively. This trend agrees with the molar mass results in Section 2.2: although increasing the AMPS content favored chain expansion, the concurrent decrease in molar mass reduced the effective hydrodynamic volume and extent of interchain entanglement [15,32,33]. Under salt-free conditions, molar mass therefore remained the principal determinant of absolute thickening capacity.

The concentration–viscosity relationships changed markedly in 1.40 g/cm^3^ potassium formate brine. As shown in Figure 4b, apparent viscosity still increased with polymer concentration, but the sample ranking differed from that in deionized water. At a polymer concentration of 0.25 wt%, the apparent viscosities of SN-1 through SN-5 were 33, 42, 48, 49, and 45 mPa·s; at 0.30 wt%, they were 44, 56, 63, 65, and 58 mPa·s, respectively. SN-4 produced the highest absolute viscosity in the target brine, closely followed by SN-3, whereas low-AMPS SN-1 was the weakest thickener. The strong hydration of AMPS sulfonate groups therefore mitigated the adverse effects of concentrated salt on chain conformation and solvation, allowing polymers with intermediate-to-high AMPS contents to retain greater thickening capacity [15,16,17,18].

Figure 4c further shows how the apparent viscosity of 0.25 wt% polymer solutions changed with brine density. As the medium density increased from 1.00 to 1.40 g/cm^3^, the apparent viscosity of SN-1 decreased from 96 to 33 mPa·s (approximately 65.6%), SN-2 from 85 to 42 mPa·s (approximately 50.6%), SN-3 from 71 to 48 mPa·s (approximately 32.4%), and SN-4 from 58 to 49 mPa·s (approximately 15.5%). In contrast, the viscosity of SN-5 changed only from 46 to 45 mPa·s and was essentially insensitive to the increase in brine density. As medium density increased, the highest-viscosity sample shifted from low-AMPS SN-1 to intermediate/high-AMPS SN-3 and SN-4, showing that higher AMPS contents substantially reduced the brine-density sensitivity of polymer thickening. Although SN-5 provided the greatest viscosity retention, its lower molar mass limited its absolute viscosity, demonstrating that high salt-tolerance retention does not necessarily produce the highest practical thickening capacity.

The storage stability of the five polymers in the target potassium formate brine is shown in Figure 4d. Viscosity retention decreased with storage time for every sample, but the decrease became progressively smaller as the AMPS content increased. After 72 h, viscosity retention values for SN-1 through SN-5 were approximately 78%, 84%, 90%, 95%, and 97%, respectively. SN-1 and SN-2 declined rapidly during the first 24 h and then gradually stabilized, whereas SN-4 and SN-5 changed only slightly over the full storage period. Increasing the AMPS content therefore improved not only hydration and dissolution in concentrated brine but also the long-term stability of the thickened structure [15,16,18].

Together, the results in Section 2.2 and Section 2.3 indicate that two competing effects control the influence of AMPS content on thickening [13,15,18,33]. On the one hand, increasing the AMPS content lowers polymer molar mass, decreasing chain entanglement and absolute thickening capacity in deionized water. On the other hand, AMPS sulfonate groups strengthen hydration and salt tolerance, allowing the polymer chains to remain relatively expanded and stably solvated in high-density potassium formate brine. Absolute viscosity in the target brine consequently increased and then decreased with AMPS content rather than changing monotonically. Within the investigated range, SN-4, with 17.9 wt% AMPS in the feed, combined high brine viscosity with approximately 95% viscosity retention after 72 h and achieved the most favorable balance among molar mass, salt tolerance, and storage stability.

### 2.5. Molecular Mechanism by Which AMPS Content Controls Polymer Performance

**Figure 5:**
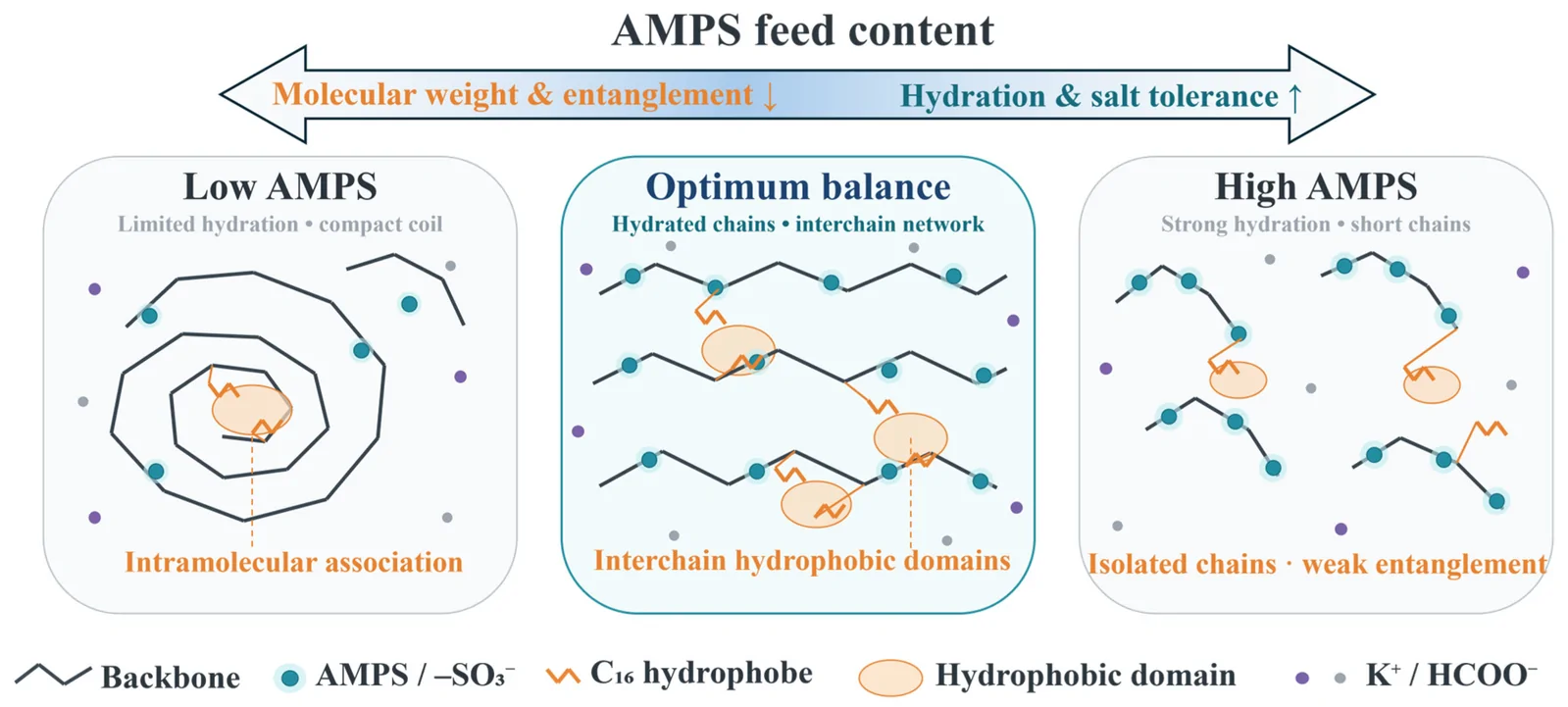
Schematic mechanism by which AMPS feed content controls polymer-chain conformation, hydrophobic association, and macroscopic performance.

The results in Section 2.2, Section 2.3 and Section 2.4 show that AMPS does not exert a uniformly beneficial effect; rather, polymer performance reflects competition between hydration and salt tolerance and molar mass and chain entanglement. As illustrated in Figure 5, when the feed content of the C_16_ hydrophobic monomer, sodium N-hexadecyl-2-acrylamidoethanesulfonate, is held constant, increasing the AMPS feed content increases the number of strongly hydrated sulfonate groups along the polymer chain and progressively improves hydration and adaptation to concentrated brine. At the same time, polymer molar mass, chain length, and interchain-entanglement capacity decrease overall. These opposing effects jointly determine dissolution, the mode of hydrophobic association, and final thickening performance in potassium formate weighted brine [15,23,28,29].

At low AMPS content, the polymers have relatively high molar masses and long chains, but too few sulfonate groups to form a sufficiently developed and stable hydration shell in 1.40 g/cm^3^ potassium formate weighted brine. Under high ionic strength, the chains readily contract into compact polymer coils, restricting solvent diffusion into the coil interior. The C_16_ hydrophobes along a given chain are then more likely to associate intramolecularly [23,24,29,31]. Such intramolecular hydrophobic association further restricts segmental expansion and reduces opportunities for effective hydrophobic junctions to form between separate chains [23,30]. Thus, despite their molar-mass and entanglement advantages, low-AMPS polymers hydrate slowly, remain insufficiently expanded in brine, and cannot form a uniform, continuous interchain associative network.

At an intermediate AMPS content, hydration of the sulfonate groups promotes solvent penetration into the polymer coils and mitigates salt-induced contraction, gradually converting compact coils into more expanded hydrated conformations. The polymer still retains sufficient molar mass, chain length, and entanglement capacity, allowing C_16_ groups on different chains to approach one another and form interchain hydrophobic microdomains [23,24,25,26]. These microdomains act as reversible physical junctions that connect multiple hydrated chains into a continuous three-dimensional network [35,36,37]. The combined action of segmental hydration and expansion, interchain entanglements, and hydrophobic association provides rapid dissolution, high absolute viscosity, and good long-term viscosity retention in potassium formate weighted brine.

At a higher AMPS content, hydration and salt tolerance improve further and the chains hydrate and dissolve rapidly. However, the excessive AMPS feed content is accompanied by lower polymer molar mass and chain length and markedly weaker interchain entanglement. With the hydrophobic-monomer feed unchanged, shorter and relatively isolated chains cannot establish a highly connected interchain hydrophobic-association network [23,28,29], and some hydrophobes form only local associative structures. Accordingly, high-AMPS samples show high viscosity retention and good brine stability, but their absolute thickening capacity is limited by lower molar mass, weaker chain entanglement, and reduced network connectivity [15,18,26]. High salt-tolerance retention therefore does not necessarily correspond to the highest absolute viscosity in brine.

The essential role of AMPS is thus to balance the hydration–salt-tolerance effect against the molar-mass–chain-entanglement effect. At insufficient AMPS content, poor hydration and intramolecular hydrophobic association restrict formation of an interchain network; at excessive AMPS content, lower molar mass and weaker chain entanglement limit absolute thickening. In SN-4, the AMPS feed content of 17.9 wt% provides a favorable combination of chain hydration and expansion, chain length, interchain entanglement, and hydrophobic-association strength, yielding the best overall performance. SN-4 was therefore selected as the representative polymer for subsequent investigation of the formation and rheology of the non-crosslinked dynamic network formed through hydrophobic association.

### 2.6. Structural Basis of the Non-Crosslinked SN-4 Dynamic Network

The preceding results show that AMPS content has a dual effect on polymer performance in potassium formate weighted brine. Increasing the AMPS content improves hydration, dissolution, and salt-tolerant stability, but concurrently lowers molar mass and interchain-entanglement capacity. SN-4, with 17.9 wt% AMPS in the feed, provided a favorable balance between these effects and exhibited both high absolute viscosity and approximately 95% viscosity retention after 72 h in 1.40 g/cm^3^ potassium formate brine. SN-4 was therefore selected to examine the structural basis and rheological response of the non-crosslinked dynamic network formed through hydrophobic association.

From the standpoint of molecular design, formation of the SN-4 network depends on the combined effects of chain hydration and expansion, interchain entanglement, and hydrophobic association. As illustrated in Figure 5, the AMPS sulfonate groups in SN-4 are strongly hydrated and mitigate the adverse effect of concentrated potassium formate on chain solvation, allowing the chains to retain a degree of expansion in the high-salinity medium. The appropriate molar mass and chain length provide a structural basis for interpenetration and entanglement among different chains. Under these conditions, long-chain C_16_ hydrophobes distributed along different polymer chains aggregate into interchain hydrophobic microdomains. These microdomains act as reversible physical junctions that connect multiple hydrated polymer chains and provide the structural basis for a continuous spatial network [23,24,25,26].

No metal or covalent crosslinker is introduced during preparation of this system. Network junctions arise mainly from reversible association among the C_16_ hydrophobes and from physical entanglement among macromolecular chains. The network therefore differs from a permanently chemically crosslinked network with fixed junctions. Under quiescent or low-shear conditions, interchain hydrophobic association junctions maintain connections among chains and form a transient network with measurable structural strength [35,36,37]. Under strong shear, some of these junctions dissociate, the chains orient along the flow direction, and the network weakens. When shear is reduced or removed, the hydrophobes can reaggregate and restore part of the network connectivity, providing potential shear responsiveness and structural recovery [26,35,36,37].

On this basis, the thickened structure formed by SN-4 in potassium formate weighted brine can be regarded as a non-crosslinked dynamic network comprising hydrated polymer chains, interchain entanglements, and reversible hydrophobic association junctions. This structure can provide viscoelasticity and proppant suspension under low shear while lowering flow resistance through temporary dissociation of associative junctions under high shear [27,35,36,37]. Network formation and dynamic reversibility were evaluated by oscillatory rheology, shear breakdown–recovery testing, pipe-flow drag reduction, and proppant-suspension experiments.

### 2.7. Viscoelasticity and Shear Recovery of the Dynamic Network

**Figure 6:**
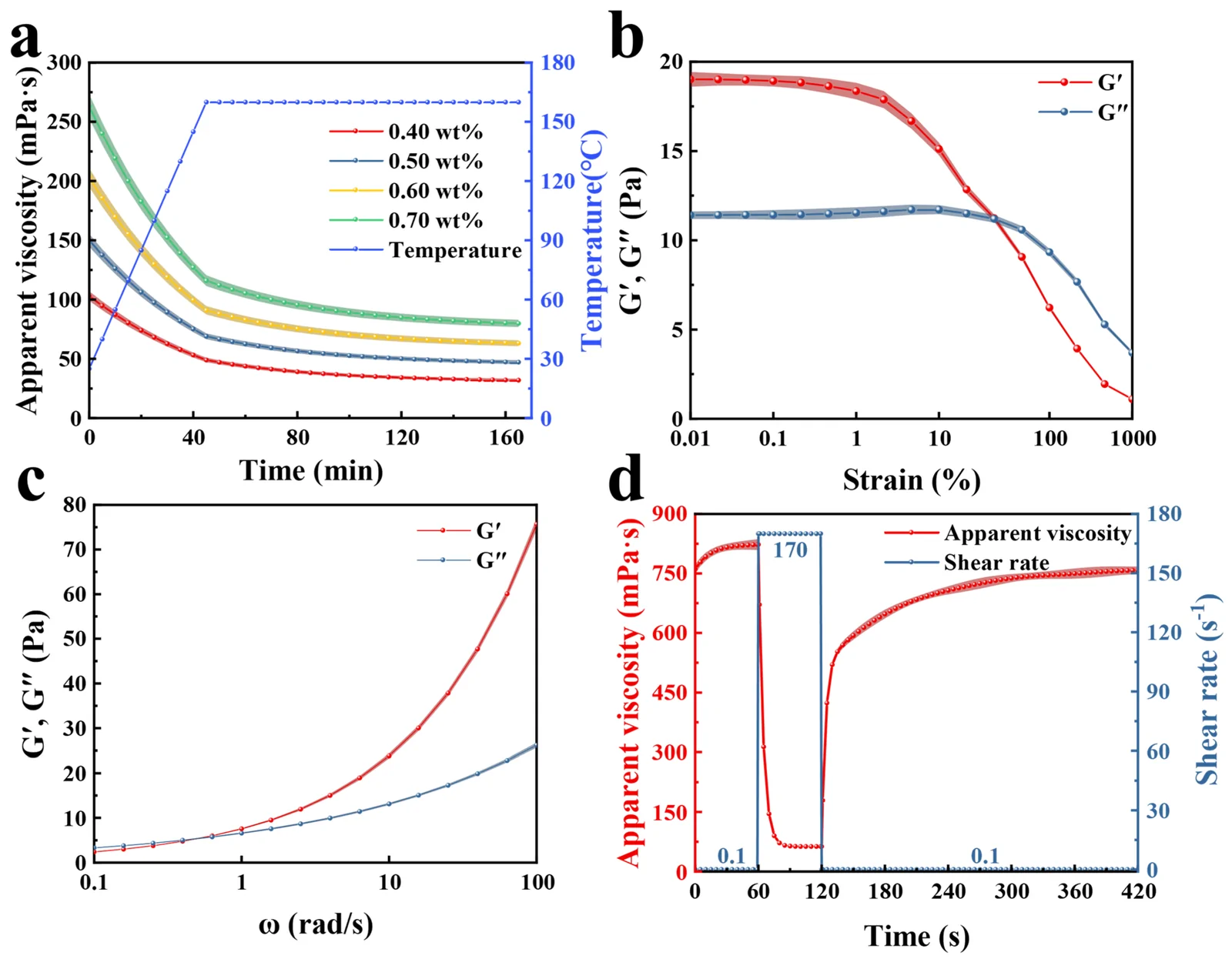
High−temperature rheology and dynamic-network behavior of SN−4 in 1.40 g/cm^3^ potassium formate weighted brine. (**a**) High−temperature continuous−shear curves for fluids containing different SN−4 concentrations; (**b**) amplitude sweep of the 0.60 wt% SN-4 fluid; (**c**) frequency sweep of the 0.60 wt% SN−4 fluid; and (**d**) apparent−viscosity response of the 0.60 wt% SN−4 fluid during a three−stage step-shear test, with the applied shear-rate program shown on the right axis. For all measured rheological quantities, solid lines represent the mean values of three replicate experiments, and the shaded regions indicate ±1 standard deviation (*n* = 3).

To determine whether the non-crosslinked network formed by SN-4 in high-temperature weighted brine can provide both viscoelastic support and post-shear reconstruction, the high-temperature shear resistance of fluids containing different polymer concentrations was first measured. Amplitude sweeps, frequency sweeps, and a three-stage shear-recovery test were then conducted at the selected concentration. The test medium was 1.40 g/cm^3^ potassium formate weighted brine, and the dynamic rheological tests were performed at 160 °C. Each experiment was repeated three times, and the results are presented as mean ± standard deviation (SD, *n* = 3). The shaded regions in Figure 6 represent ±1 SD.

As shown in Figure 6a, under a shear rate of 170 s^−1^, the apparent viscosities of the fluids containing different SN-4 concentrations decreased substantially as the temperature increased from 25 to 160 °C. After the temperature reached 160 °C, the rate of viscosity decline gradually decreased and each curve approached a plateau. At the end of the 120 min isothermal-shearing stage, the apparent viscosities of the 0.40, 0.50, 0.60, and 0.70 wt% SN-4 fluids were 32.0 ± 1.3, 47.2 ± 1.7, 63.2 ± 2.5, and 79.9 ± 3.1 mPa·s, respectively. The corresponding relative standard deviations ranged from 3.6% to 4.0%, indicating good reproducibility of the high-temperature continuous-shear measurements. Higher polymer concentrations increased the high-temperature viscosity plateau, reflecting an increase in the density of interchain entanglements and hydrophobic-association junctions. Using a final viscosity of at least 50 mPa·s after continuous shearing at 160 °C as the selection criterion, the 0.40 and 0.50 wt% fluids did not qualify, whereas the 0.60 and 0.70 wt% fluids retained higher residual viscosities. Considering both high-temperature shear resistance and polymer dosage, 0.60 wt% was selected for the subsequent dynamic rheological tests.

Figure 6b shows the amplitude sweep of the 0.60 wt% SN-4 fluid at 160 °C and an angular frequency of 6.28 rad/s. At low strain, within the linear viscoelastic region (LVR), the storage modulus (G′) and loss modulus (G″) remained essentially constant at plateau values of approximately 19.0 ± 0.4 and 11.4 ± 0.3 Pa, respectively. The loss tangent (tan δ) was approximately 0.60, and G′ remained above G″, indicating predominantly elastic behavior under small deformation [38,39]. Using a 10% decrease in G′ from its low-strain plateau as the criterion, the critical strain (γ_c_) was approximately 3.9 ± 0.3%. As strain increased further, G′ decreased much more rapidly than G″, and the moduli crossed at approximately 30 ± 0.3% strain. Above the crossover, G″ exceeded G′, indicating that large deformation disrupted the original network connections and changed the response from elasticity-dominated to viscous-flow-dominated [35,37,38]. This strain dependence shows that the SN-4 network has finite structural strength and undergoes dissociation of hydrophobic associations and shear-induced orientation and slippage under large deformation [35,36,37,38].

The frequency sweep at 1% strain is shown in Figure 6c. In the low-frequency region, G″ exceeded G′; at 0.1 rad/s, for example, G′ and G″ were approximately 2.4 ± 0.05 and 3.30 ± 0.06 Pa, respectively. Thus, over long observation times, the network junctions could dissociate and rearrange, producing a strong viscous contribution. As angular frequency increased, G′ rose more rapidly than G″, and the moduli crossed at approximately 0.50 rad/s. At 100 rad/s, G′ and G″ reached approximately 75.7 ± 1.3 and 26.2 ± 0.6 Pa, respectively, and the response became elasticity-dominated. The characteristic relaxation time (τ) estimated from the crossover angular frequency (ω_c_) was τ ≈ 1/ω_c_ ≈ 2.0 s. The network therefore has a finite lifetime: it stores deformation energy over short time scales but relaxes stress through dissociation and reorganization of physical junctions over longer time scales. This frequency dependence is characteristic of a transient physical network [35,36,37].

The three-stage shear test further confirmed network recoverability (Figure 6d). During the first stage at 0.1 s^−1^, the apparent viscosity gradually stabilized at approximately 823 ± 14.6 mPa·s. Increasing the shear rate to 170 s^−1^ rapidly reduced the apparent viscosity to a final value of approximately 63 ± 0.7 mPa·s, representing a decrease of approximately 92.3 ± 0.1%, showing that high shear strongly disrupted chain entanglements and dynamic physical junctions. When the shear rate was returned to 0.1 s^−1^, the apparent viscosity recovered rapidly at first and then more gradually. After 60 s, the viscosity was approximately 647 ± 11.0 mPa·s, corresponding to a recovery ratio of approximately 78.7 ± 0.1%; after 300 s, it reached approximately 758 ± 9.5 mPa·s, corresponding to a final recovery ratio of approximately 92.1 ± 0.5%. The recovery ratio was calculated relative to the viscosity at the end of the first stage. Recovery of most of the initial viscosity within a short period shows that network breakdown under high shear was dominated by reversible physical dissociation [26,35,36,37]. The small unrecovered fraction may reflect incomplete equilibration or limited irreversible chain damage.

Taken together, the amplitude-sweep, frequency-sweep, and shear-recovery results show that SN-4 formed a dynamic physical network with a finite relaxation time, strain sensitivity, and substantial reconstruction capacity in high-temperature potassium formate weighted brine [35,36,37,38]. This behavior agrees with the proposed structure of hydrated polymer chains, interchain entanglements, and reversible hydrophobic association junctions. Under low shear or high-frequency perturbation, entanglements and associative junctions jointly impart elasticity. Under high shear, the junctions dissociate and the network breaks down, reducing flow resistance. As shear weakens, the hydrophobes reassociate and the network progressively reconstructs [35,36,37]. The 0.60 wt% SN-4 fluid therefore combines high-temperature shear resistance with post-shear recovery at 160 °C, providing a rheological basis for recirculation and stable proppant transport in a non-chemically crosslinked weighted fracturing fluid.

### 2.8. Contribution of the Dynamic Network to Drag Reduction and Proppant Suspension

**Figure 7:**
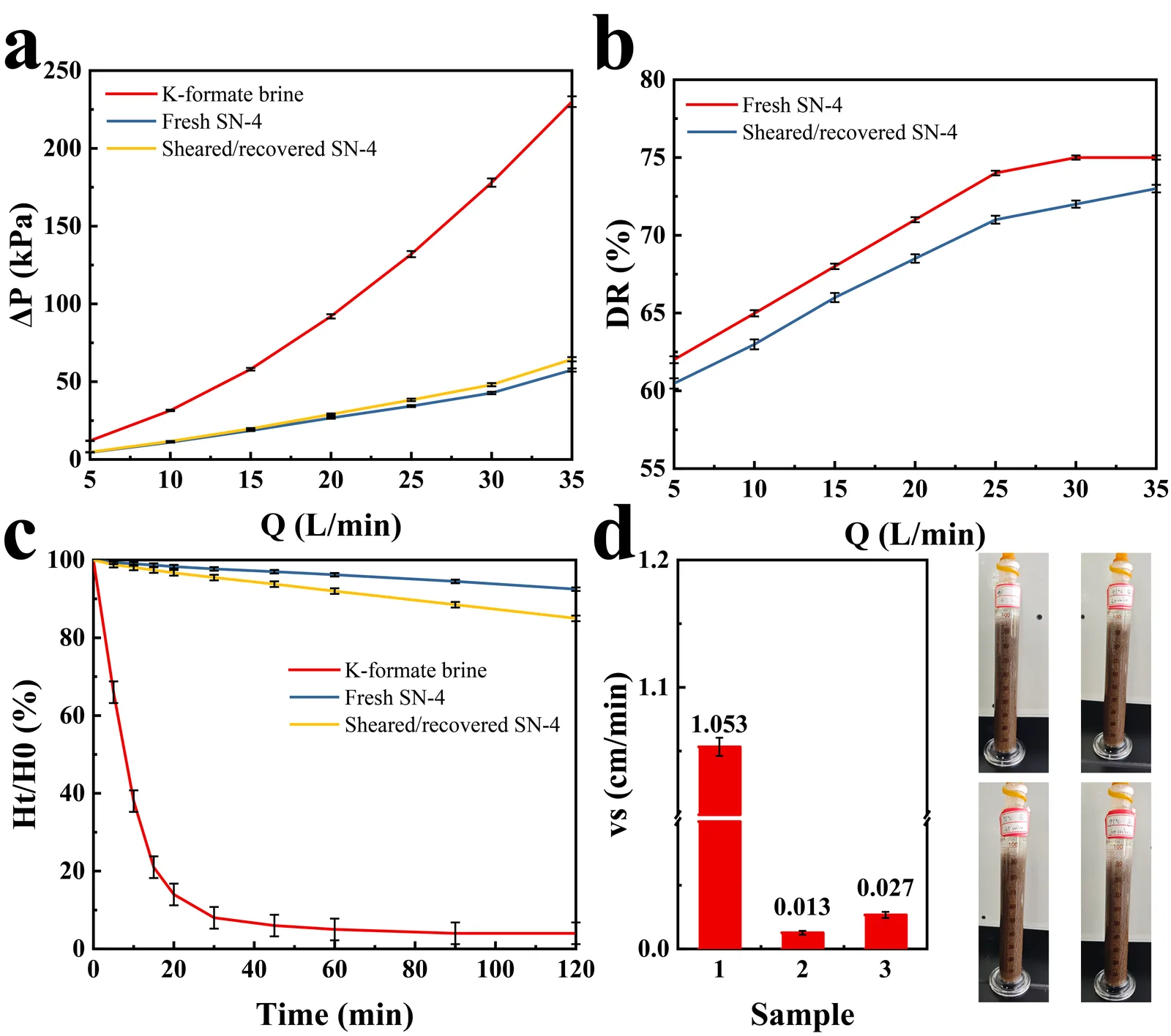
Drag-reduction and static proppant-suspension performance of SN-4 weighted fracturing fluids. (**a**) Pressure drop across the test section at different flow rates for potassium formate brine, freshly prepared SN-4 fluid, and thermally sheared–recovered SN-4 fluid; (**b**) drag-reduction rates of fresh and recovered SN-4 fluids; (**c**) normalized proppant-suspension height as a function of time; and (**d**) mean proppant settling velocities and representative static-suspension photographs for the different fluids. Symbols and bars represent the mean values of three independent measurements, and error bars indicate ±1 SD (*n* = 3).

To evaluate the contribution of the dynamic network to drag reduction, pressure drops across the test section were measured at different flow rates for blank potassium formate brine, freshly prepared SN-4 fluid, and thermally sheared–recovered SN-4 fluid. As shown in Figure 7a, increasing the flow rate from 5 to 35 L/min raised the pressure drop of blank potassium formate brine nonlinearly from approximately 12 to 230 kPa. In contrast, the pressure-drop increase was strongly suppressed in the fresh and recovered SN-4 fluids, which reached only approximately 58 and 62 kPa, respectively, at 35 L/min. At 30 L/min, the blank-brine pressure drop was approximately 178 kPa, whereas those of the fresh and recovered SN-4 fluids were only approximately 45 and 50 kPa. The dynamic network formed by SN-4 therefore substantially reduced pipe friction at high flow rates.

As shown in Figure 7b, over flow rates of 5–35 L/min, the drag-reduction rate of freshly prepared SN-4 increased from approximately 62% to 75%, whereas that of the thermally sheared–recovered fluid increased from approximately 60.5% to 73%. Both states maintained drag-reduction rates above 60%. With increasing flow rate, drag reduction first increased markedly and then approached a plateau at 30–35 L/min, indicating progressive chain extension under stronger flow shear and effective suppression of turbulent vortices and radial momentum transfer [19,20,21,22]. The recovered fluid showed drag-reduction rates only approximately 2–3 percentage points lower than those of the fresh fluid; at 30 L/min, its post-shear drag-reduction retention was approximately 96%. This result is consistent with the 92.1% viscosity recovery measured 300 s after high shear. Although high-temperature shearing caused some chain damage and dissociation of associative structures, the remaining hydrophobic units could reassociate as shear weakened, restoring the dynamic network and maintaining high drag-reduction performance [26,35,36].

The dynamic network also markedly improved static proppant suspension. As shown in Figure 7c, the proppant settled rapidly in potassium formate brine: the normalized suspension height, $H_{t}/H_{0}$, where $H_{0}$ is the initial suspension-layer height and $H_{t}$ is the height at time t, decreased to approximately 21% after 15 min, approximately 5% after 60 min, and a plateau of approximately 4% after 120 min. The suspension layer in the fresh SN-4 fluid decreased slowly, retaining approximately 96% and 92% of its initial height at 60 and 120 min, respectively. After high-temperature shearing and recovery, the corresponding values remained approximately 92% and 85%. The mean apparent proppant settling velocities in potassium formate brine, fresh SN-4 fluid, and recovered SN-4 fluid were 1.053, 0.013, and 0.027 cm/min, respectively (Figure 7d). Relative to blank brine, the settling velocities in the two SN-4 fluids were reduced by approximately 98.8% and 97.4%, showing that the reconstructed network retained a strong ability to constrain particles.

The graduated-cylinder photographs recorded from 15 to 60 min in Figure 7d further support these results. The proppant remained generally well dispersed during the observation period, with no obvious upper clear-fluid layer or sharply defined settling interface; only a slight vertical concentration gradient appeared at later times. The viscoelastic network formed by SN-4 therefore delayed gravitational settling of the dense proppant by increasing continuous-phase flow resistance, restricting relative particle motion, and providing elastic support [40,41,42]. The somewhat greater settling velocity after thermal shear indicates that limited chain scission and lower network density weakened suspension, but reversible hydrophobic association still maintained performance far above that of blank brine [43,44].

Overall, the SN-4 dynamic network performs two coordinated functions under different flow conditions. Under high-shear pipe flow, extended polymer chains suppress turbulent fluctuations and reduce friction [19,20,21,22]. Under low shear or quiescent conditions, a three-dimensional network formed by chain entanglement and reversible hydrophobic association limits proppant settling [40,41,42]. Even after high-temperature shearing, the fluid maintained a drag-reduction rate above 60% and a low settling velocity. Its rapid structural recovery, pipe-flow drag reduction, and static proppant stability provide a basis for low-friction transport and stable proppant placement in high-temperature, high-salinity reservoir stimulation.

## 3. Conclusions

AM/AMPS/sodium N-hexadecyl-2-acrylamidoethanesulfonate hydrophobically associating polymers were prepared, and the effects of AMPS feed content on molecular structure, hydration in concentrated brine, thickening, and the rheology of a non-crosslinked dynamic network were systematically examined. Drag reduction and proppant suspension in potassium formate weighted fracturing fluids were also evaluated. The principal conclusions are as follows.

(1) With the hydrophobic-monomer feed and degree of hydrolysis held essentially constant, increasing the AMPS feed content simultaneously decreased polymer molar mass and increased chain expansion. As the AMPS feed content increased from 5.5 to 23.8 wt%, M_w_ decreased from 25.83 × 10^6^ to 9.26 × 10^6^ g/mol, M_n_ decreased from 14.35 × 10^6^ to 3.63 × 10^6^ g/mol, and the mean R_g_ decreased from 350.97 to 244.94 nm, whereas the chain-conformation exponent increased from 0.570 to 0.610. Under the present synthesis conditions, higher AMPS content increased chain expansion at a given molar mass, but the decrease in molar mass still reduced overall coil dimensions.

(2) AMPS units substantially improved polymer hydration, dissolution, and storage stability in concentrated potassium formate brine. As the AMPS feed content increased from 5.5 to 23.8 wt%, t_90_ in 1.40 g/cm^3^ potassium formate brine decreased from approximately 118 to 48 min, and the brine-induced hydration delay became much smaller. Absolute thickening in brine did not increase monotonically with AMPS content, however, because it was governed by competition between salt-tolerant hydration and molar mass and chain entanglement. SN-4, with 17.9 wt% AMPS in the feed, reached apparent viscosities of 49 and 65 mPa·s at polymer concentrations of 0.25 and 0.30 wt%, respectively, and retained approximately 95% of its viscosity after 72 h in the target brine, providing a favorable balance among molar mass, salt tolerance, and thickening stability.

(3) A 0.60 wt% SN-4 fluid in 1.40 g/cm^3^ potassium formate brine exhibited favorable high-temperature viscoelasticity and post-shear recovery. Its final viscosity remained approximately 63 mPa·s after continuous shearing at 160 °C and 170 s^−1^. In the LVR, G′ exceeded G″, and the critical strain was approximately 3.9%. The crossover angular frequency was approximately 0.50 rad/s, corresponding to a characteristic relaxation time of approximately 2.0 s. In the three-stage shear test, viscosity recovery ratios after 60 and 300 s were 78.7% and 92.1%, respectively. These results support a model of a non-crosslinked transient network maintained by hydrated polymer chains, interchain entanglements, and reversible hydrophobic association junctions; the network dissociates under high shear and rapidly reconstructs as shear weakens.

(4) The non-crosslinked SN-4 dynamic network combined drag reduction at high flow rates with proppant suspension under low shear. Over 5–35 L/min, both fresh and thermally sheared–recovered fluids maintained drag-reduction rates above 60%; at 30 L/min, the recovered fluid retained approximately 96% of the fresh-fluid drag reduction. In static-suspension tests, the fresh and recovered fluids retained approximately 92% and 85% of their initial suspension heights after 120 min and exhibited mean proppant settling velocities of 0.013 and 0.027 cm/min, representing reductions of 98.8% and 97.4% relative to blank potassium formate brine. This shear-responsive network therefore enables low friction under high shear and stable proppant suspension under low shear, providing a feasible route to non-chemically crosslinked weighted fracturing fluids for high-temperature, high-salinity service.

## 4. Materials and Methods

### 4.1. Materials

The reagents, grades, and suppliers used in this study are listed in Table 2. Reagents were used without further purification. Unless otherwise specified, all water used throughout this study was produced in-house using an arium^®^ ultrapure water purification system (Sartorius Lab Instruments GmbH & Co. KG, Göttingen, Germany) and had a resistivity greater than 18.0 MΩ·cm at 25 °C. For consistency, this water is referred to as “deionized water” throughout the manuscript.

The normal boiling points or relevant thermal behavior of the reagents are also summarized in Table 2. For nonvolatile ionic solids and thermally unstable compounds that undergo decomposition or polymerization before boiling, a conventional boiling point is not applicable; these substances are therefore identified as “N/A” together with their relevant thermal behavior.

### 4.2. Experimental Methods

Unless otherwise specified, all concentrations are expressed as mass fractions relative to the total solution mass. Replicate measurements and the corresponding statistical treatment are specified in the relevant experimental sections and figure captions. All instruments were calibrated according to the manufacturers’ instructions before use.

The principal instruments and devices used in this study, together with their models, manufacturers, locations, and applications, are summarized in Table 3. Laboratory-built apparatuses are identified accordingly, and their key dimensions and operating conditions are described in the corresponding experimental subsections.

#### 4.2.1. Polymer Preparation

**Figure 8:**
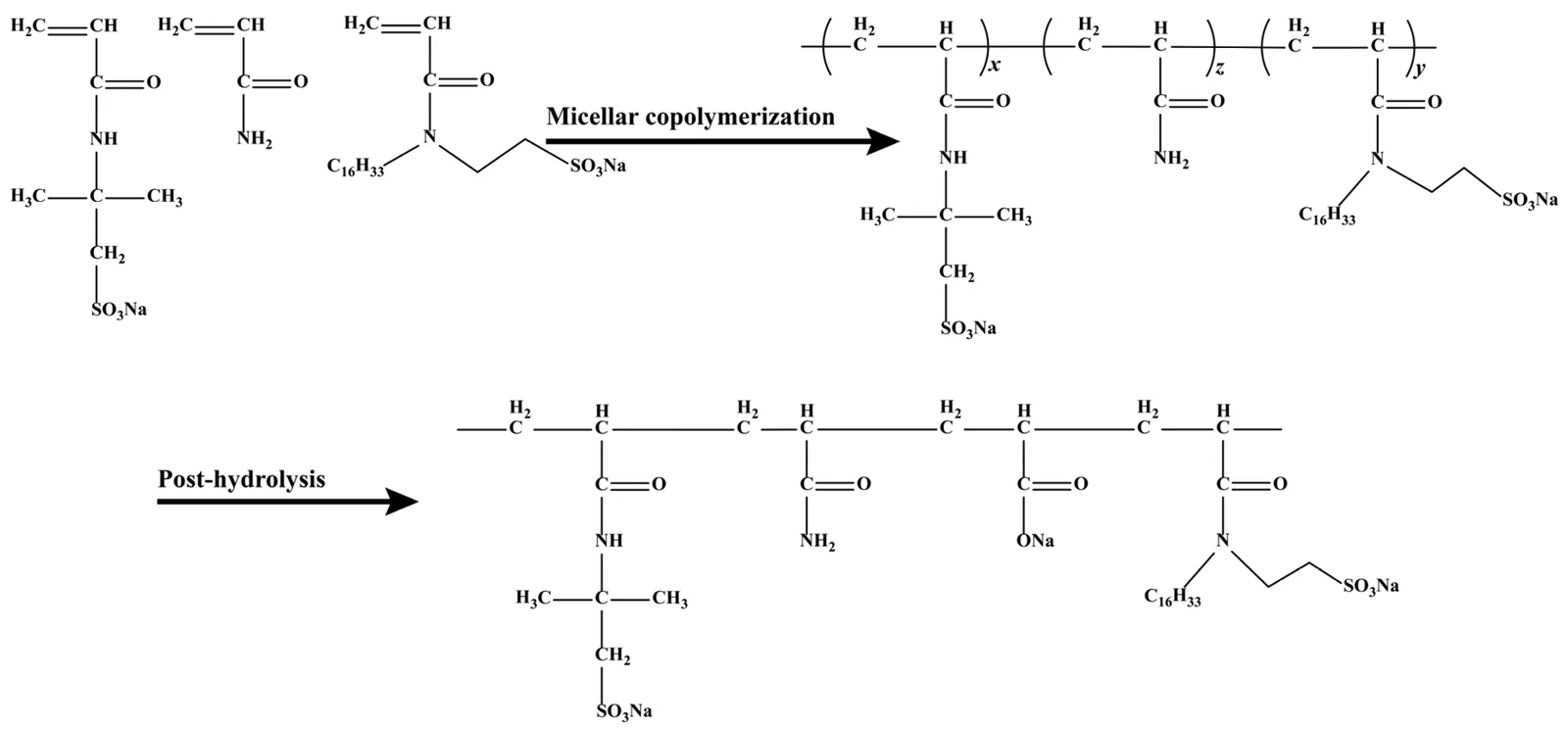
Low-temperature micellar copolymerization followed by post-hydrolysis for preparation of the hydrophobically associating polymers.

Model polymers with different AMPS contents were synthesized by low-temperature micellar copolymerization using a combined redox/azo initiation system, followed by alkaline post-hydrolysis, as schematically illustrated in Figure 8. For SN-1–SN-5, the feed fraction of sodium N-hexadecyl-2-acrylamidoethanesulfonate was fixed at 0.4 wt%, whereas the AMPS feed fractions were 5.5, 9.0, 13.0, 17.9, and 23.8 wt%, respectively. Acrylamide accounted for the balance of the monomer feed, corresponding to 94.1, 90.6, 86.6, 81.7, and 75.8 wt%, respectively. All monomer feed fractions were calculated relative to the total monomer mass before post-hydrolysis.

Acrylamide, AMPS, and the hydrophobic monomer were added to deionized water and dissolved or dispersed using a laboratory overhead mechanical stirrer operated at 300 rpm until a homogeneous monomer solution was obtained. The total monomer concentration in the reaction mixture was maintained at 25 wt%. Following the mildly alkaline preparation conditions used for a related AM/AMPS/C16 associative polymer system [24], the pH was adjusted to 8.0 using sodium hydroxide and acetic acid. Sodium formate was subsequently added as a molar-mass regulator (chain-transfer agent) at 0.050 wt% relative to the total monomer mass.

The reaction vessel containing the monomer solution was placed in a conventional chest freezer, and the solution temperature was monitored directly using a laboratory thermometer. Cooling was continued until the internal temperature of the solution reached 0 °C. The vessel was then removed from the freezer, and nitrogen was bubbled through the solution for 30 min while mechanical stirring was maintained at 300 rpm to remove dissolved oxygen.

Freshly prepared 1.0 wt% aqueous solutions of sodium bisulfite (NaHSO_3_), ammonium persulfate (APS), and 2,2′-azobis[2-(2-imidazolin-2-yl) propane] dihydrochloride (VA-044) were subsequently added, followed by tert-butyl hydroperoxide (TBHP). Their active dosages were 0.020, 0.020, 0.020, and 0.005 wt%, respectively, relative to the total monomer mass, corresponding to a total active initiator dosage of 0.065 wt%. TBHP was used as a 70 wt% aqueous solution, and its added mass was corrected according to the active TBHP content. The water used to prepare the initiator solutions was included in the total water mass used to obtain the prescribed monomer concentration. After addition of TBHP, polymerization was allowed to proceed under a nitrogen atmosphere in a thermally insulated reaction vessel for 4 h without external heating.

After polymerization, the resulting polymer gel was granulated into particles of approximately 2–5 mm. For post-hydrolysis, the NaOH dosage was calculated according to a molar ratio of $n_{\mathrm{NaOH}}/n_{\mathrm{AM}}=0.30$, corresponding to a target degree of hydrolysis of approximately 30 mol%. The active NaOH dosages for SN-1, SN-2, SN-3, SN-4, and SN-5 were 15.89, 15.30, 14.62, 13.79, and 12.80 g per 100 g of total monomers, respectively. NaOH was added as a 30 wt% aqueous solution and uniformly mixed with the granulated polymer gel. Post-hydrolysis was conducted at 60 °C for 4 h.

The hydrolyzed products were vacuum-dried at 60 °C for 24 h or until a constant mass was obtained, pulverized, and passed through an 80-mesh sieve, corresponding to a nominal particle size below approximately 180 μm. The experimentally measured degrees of hydrolysis of the resulting polymers ranged from 29.1% to 31.2%, as summarized in Table 1.

#### 4.2.2. Preparation of Potassium Formate Brines and Polymer Solutions

Potassium formate brines of different concentrations were prepared gravimetrically, and their densities were measured at 20 °C using a buoyancy densimeter. By varying the potassium formate content, brines with densities of 1.10, 1.20, 1.30, and 1.40 g/cm^3^ were obtained.

To prepare a polymer solution, the polymer powder was added slowly to deionized water or potassium formate brine under stirring at 300 r/min to avoid agglomeration. The solutions were considered fully hydrated when the apparent viscosity changed by less than 5% over 30 consecutive min. Except in the hydration-kinetics experiments, fully hydrated solutions were used for all performance tests. Polymer concentration is reported as the mass fraction of polymer powder in the total solution.

#### 4.2.3. Degree of Hydrolysis and Chemical-Structure Characterization

The degree of hydrolysis was measured in accordance with GB/T 12005.6-1989, determination of hydrolysis degree of partly hydrolysis polyacrylamide [45].

Polymer chemical structure was characterized by ^1^H NMR. Purified and fully dried polymer was dissolved in deuterium oxide (D_2_O) and analyzed using a 600-MHz Bruker Avance NEO NMR spectrometer following the general protocol for NMR analysis. Polymer structure was qualitatively confirmed from the chemical shifts in characteristic proton signals.

#### 4.2.4. Molar Mass and Chain-Conformation Measurements

The weight-average molar mass (M_w_), number-average molar mass (M_n_), dispersity (Đ), and radius of gyration (R_g_) were measured by size-exclusion chromatography coupled with multi-angle light scattering (SEC–MALS) [32]. The system comprised a DAWN HELEOS II multi-angle light-scattering detector, a G6000PWXL column, and an Optilab T-rEX differential refractometer serving as the differential refractive-index detector.

The sample-preparation procedure followed the method reported by Woo et al. [32]. The polymer powders were dissolved in deionized water at a concentration of 0.5 mg/mL. The solutions were initially stirred at 300 r/min for 2 h using a glass-blade impeller installed on a WiseStir HS-30E mechanical stirrer (Daihan Scientific Co., Wonju, Republic of Korea), after which the stirring rate was reduced to 150 r/min and maintained for an additional 22 h to ensure complete polymer hydration while minimizing shear-induced chain degradation. Sodium nitrate was subsequently added to obtain a final NaNO_3_ concentration of 0.1 mol/L, and the solutions were further stirred at 300 r/min for 30 min.

The polymer solutions were analyzed without centrifugation or membrane filtration. Membrane filtration was deliberately avoided because it could selectively retain ultrahigh-molar-mass polymer chains and hydrophobically associated species, thereby biasing the measured molar-mass distribution. Accordingly, the reported SEC–MALS results represent the complete water-dispersible polymer fractions rather than prefiltered soluble fractions. The mobile phase consisted of deionized water containing 0.1 mol/L sodium nitrate (NaNO_3_) and 0.02% sodium azide (NaN_3_). Before use, the mobile phase was filtered through a 0.1 μm Durapore polyvinylidene fluoride membrane (Merck Millipore, Darmstadt, Germany). A polyether ether ketone (PEEK) inline filter fitted with a 0.1 μm VVLP membrane was installed downstream of the pump to prevent particulate contamination of the analytical system.

The refractive-index increment (dn/dc) of each polymer was independently measured using the Optilab T-rEX differential refractometer, and the corresponding measured value was used for the molar-mass calculation.

Dispersity was calculated as follows:

(1) Đ = M_w_/M_n_

Within the common molar mass range covered by all samples and having a reliable signal-to-noise ratio, R_g_ was fitted against molar mass M according to Equation (2):

(2) R_g_ = KM^ν^

where K is a proportionality constant and ν is the chain-conformation exponent. Linear regression of log Rg against log M was used to determine ν.

#### 4.2.5. Polymer Hydration and Dissolution Kinetics

The hydration behavior of SN-1 through SN-5 was compared in deionized water and 1.40 g/cm^3^ potassium formate brine at identical polymer concentration, particle size, addition procedure, stirring rate, and temperature. Polymer concentration was 0.4 wt%, stirring rate was 300 r·min^−1^, and test temperature was 25 °C. Timing began when the polymer was added, and the apparent viscosity was measured over 0–180 min.

Apparent viscosity was measured with a HAAKE MARS 60 rheometer at 25 °C and 170 s^−1^. The viscosity-based degree of hydration, H_η_(t), was calculated as follows:

(3) H_η_(t) = (η_t_ − η_b_)/(η_∞_ − η_b_) × 100%

where η_t_ is the apparent viscosity at hydration time t; η_b_ is the apparent viscosity of the blank medium under the same conditions; and the plateau viscosity, η_∞_, is the viscosity of the fully hydrated polymer solution. When the viscosity changed by less than 5% over 30 consecutive min, η_∞_ was taken as the mean of the final three measurements. The hydration time t_90_ was defined as the time required to reach 90% hydration and was calculated by linear interpolation between adjacent measurement points.

In parallel, swelling and disappearance of polymer particles were recorded with a KEYENCE VK-X1000 3D laser scanning microscope at identical magnification, exposure, and imaging distance. Uniform threshold segmentation was applied to the images using ImageJ software (version 1.54p; National Institutes of Health, Bethesda, MD, USA) [46], and the residual undissolved-particle area fraction was calculated using Equation (4):

(4) RA_t_ = A_t_/A_0_ × 100%

where A_0_ is the initial projected particle area and A_t_ is the residual projected particle area at hydration time t.

#### 4.2.6. Thickening, Brine-Density Sensitivity, and Storage Stability

Polymer solutions with mass fractions of 0.05, 0.10, 0.15, 0.20, 0.25, and 0.30 wt% were prepared in deionized water and 1.40 g/cm^3^ potassium formate brine. After full hydration, apparent viscosity was measured with a HAAKE MARS 60 rheometer at 160 °C and 170 s^−1^ to compare the thickening capacity of polymers with different AMPS contents.

To evaluate the effect of brine density on thickening, 0.25 wt% polymer solutions were prepared in media with densities of 1.00, 1.10, 1.20, 1.30, and 1.40 g/cm^3^; the 1.00 g/cm^3^ medium was deionized water. After full hydration, the apparent viscosities of all solutions were measured under identical conditions. The viscosities of the corresponding blank media were also measured to exclude the contribution from the intrinsic viscosity change of potassium formate brine.

For storage-stability testing, 0.25 wt% polymer solutions were prepared in 1.40 g/cm^3^ potassium formate brine, sealed, and stored at 25 °C for 72 h. Apparent viscosity was measured at predetermined times, and the viscosity retention R_t_ was calculated as follows:

(5) R_t_ = η_t_/η_0_ × 100%

where η_0_ is the apparent viscosity at the beginning of storage and η_t_ is the apparent viscosity after storage time t.

#### 4.2.7. Continuous High-Temperature Shearing and Oscillatory Rheological Testing

Samples were heated from 25 to 160 °C at 3.5 °C·min^−1^ under a constant shear rate of 170 s^−1^ and then sheared isothermally at 160 °C for 120 min. Apparent viscosity and temperature were recorded continuously, and the viscosity at the end of the test was defined as the final viscosity. A final viscosity of at least 50 mPa·s after continuous shearing at 160 °C was used as the concentration-selection criterion.

Oscillatory rheological testing was conducted on a 0.60 wt% SN-4 solution. A strain sweep was first performed at 160 °C and an angular frequency of 6.28 rad/s over a strain range of 0.01–1000%, while the storage modulus (G′) and loss modulus (G″) were recorded. The strain at which G′ decreased by 10% relative to its low-strain plateau was taken as the upper limit of the linear viscoelastic region (LVR) [38,39].

A frequency sweep was then performed at 160 °C and a constant strain of 1% within the LVR over an angular-frequency range of 0.1–100 rad/s. The angular frequency at G′ = G″ was defined as the crossover angular frequency (ω_c_), and the characteristic relaxation time (τ) was estimated as follows [36,37,38,39]:

(6) τ ≈ 1/ω_c_

All continuous high-temperature shearing, amplitude-sweep, and frequency-sweep experiments were independently repeated three times. At each measurement point, the mean value and sample standard deviation were calculated, and the results are presented as mean ± SD (*n* = 3).

#### 4.2.8. Shear Breakdown–Recovery Testing

The shear breakdown–recovery behavior of the non-crosslinked SN-4 network was evaluated using a three-stage shear protocol. A 0.60 wt% SN-4 solution was heated to 160 °C and held for 10 min. In stage I, the fluid was sheared at 0.1 s^−1^ for 60 s to characterize the initial network under low shear. In stage II, it was sheared at 170 s^−1^ for 60 s to disrupt chain entanglements and hydrophobic associations. In stage III, the shear rate was returned to 0.1 s^−1^ for 300 s while viscosity recovery was recorded. All rheological measurements at 160 °C were conducted using a sealed, pressure-rated measuring cell, which prevented solvent evaporation and maintained the aqueous samples in the liquid state throughout the tests.

The viscosity recovery ratios at 60 and 300 s were calculated using Equations (7) and (8), respectively:

(7) R_η,60_ = η_III,60_/η_I,end_ × 100%

(8) R_η,300_ = η_III,end_/η_I,end_ × 100%

where η_I,end_ is the apparent viscosity at the end of stage I, η_III,60_ is the apparent viscosity after 60 s of recovery in stage III, and η_III,end_ is the apparent viscosity after 300 s of recovery in stage III.

The three-stage shear breakdown–recovery test was independently repeated three times. The viscosity decrease and recovery ratios were calculated separately for each replicate and subsequently summarized as mean ± SD (*n* = 3).

#### 4.2.9. Pipe-Flow Drag-Reduction Testing

The pipe-flow drag-reduction performance of the SN-4 weighted fracturing fluid was evaluated using a laboratory flow-loop apparatus. The test pipe had an internal diameter of 10 mm and an effective test-section length of 3 m. Pipe-flow experiments were performed at 25 °C.

The test media were blank potassium formate brine with a density of 1.40 g/cm^3^, freshly prepared 0.60 wt% SN-4 solution, and SN-4 solution that had been sheared at 160 °C and 170 s^−1^ for 120 min and then allowed to recover for 300 s. Flow rates were 5, 10, 15, 20, 25, 30, and 35 L/min. At each flow rate, the pressure drop across the test section was recorded after it had remained stable for 10 s. Each condition was tested in triplicate.

The drag-reduction rate (DR) was calculated as follows:

(9) DR = (ΔP_0_ − ΔP_p_)/ΔP_0_ × 100%

where ΔP_0_ is the pressure drop of blank potassium formate brine at the same flow rate and ΔP_p_ is that of the polymer solution. Post-shear drag-reduction retention (R_DR_) was calculated using Equation (10):

(10) R_DR_ = DR_after_/DR_fresh_ × 100%

where DR_fresh_ and DR_after_ are the drag-reduction rates of the fresh fluid and the thermally sheared–recovered fluid, respectively, at the same flow rate.

#### 4.2.10. Static Proppant-Suspension Testing

The static proppant-suspension performance of the SN-4 weighted fracturing fluid was evaluated by a static settling method [40,41,42]. A 40/70-mesh quartz-sand proppant was added at 180 kg·m^−3^ to blank 1.40 g/cm^3^ potassium formate brine, fresh 0.60 wt% SN-4 solution, and thermally sheared–recovered SN-4 solution. After uniform mixing under identical conditions, each suspension was rapidly transferred into a 20 mL sealed, transparent, pressure-rated borosilicate-glass vessel and held quiescent at 160 °C.

The initial proppant-suspension-layer height H_0_ was recorded, and the layer height H_t_ was measured over 0–120 min. The normalized proppant-suspension height (H_t_/H_0_) was calculated as follows:

(11) R_H_(t) = H_t_/H_0_ × 100%

During the interval in which the settling interface changed approximately linearly with time, the apparent proppant settling velocity (v_s_) was calculated using Equation (12):

(12) v_s_ = (H_t1_ − H_t2_)/(t_2_ − t_1_)

where H_t1_ and H_t2_ are the suspension-layer heights at times t_1_ and t_2_, respectively. Intervals after the suspension height had entered a stable plateau were excluded from the settling-velocity fit. The same vessel dimensions, proppant concentration, initial liquid-column height, and imaging conditions were used for all samples.

## Figures and Tables

**Table 1:** Monomer feed compositions, measured degrees of hydrolysis, and weight-average molar masses of the model polymers.

Sample	Hydrophobic Monomer Content (%)	Acrylamide Feed (wt %)	AMPS Feed (wt %)	Degree of Hydrolysis (mol%)	M_w_ (10^6^ g/mol)
SN-1	0.4	94.1	5.5	31.2	25.83
SN-2	0.4	90.6	9.0	30.2	21.73
SN-3	0.4	86.6	13.0	30.3	16.82
SN-4	0.4	81.7	17.9	29.4	12.69
SN-5	0.4	75.8	23.8	29.1	9.26

**Table 2:** Experimental reagents and suppliers.

Reagent	Supplier	Grade	Normal Boiling Point/Thermal Behavior
Acrylamide	Jiangxi Changjiu Biochemical Co., Ltd., Nanchang, China	Chemical grade	N/A; polymerizes or decomposes before boiling at atmospheric pressure
2-Acrylamido-2-methylpropanesulfonic acid (AMPS)	Weifang Aorui Environmental Protection Technology Co., Ltd., Weifang, China	Chemical grade	N/A; decomposes before boiling
Sodium N-hexadecyl-2-acrylamidoethanesulfonate	Sichuan Guangya Polymer Chemical Co., Ltd., Nanchong, China	Chemical grade	N/A; nonvolatile ionic solid that decomposes on heating
Sodium hydroxide	Chengdu Kelong Chemical Co., Ltd., Chengdu, China	Analytical grade	1388 °C
Glacial acetic acid	Chengdu Kelong Chemical Co., Ltd., Chengdu, China	Analytical grade	117.9 °C
Potassium formate	Xinjiang Guolin New Materials Co., Ltd., Shihezi, China	Industrial grade	N/A; nonvolatile ionic solid that decomposes before boiling
Sodium formate	Luxi Chemical Group Co., Ltd., Liaocheng, China	Industrial grade	N/A; decomposes before boiling
Sodium bisulfite	Chengdu Kelong Chemical Co., Ltd., Chengdu, China	Analytical grade	N/A; decomposes before boiling
Ammonium persulfate	Chengdu Kelong Chemical Co., Ltd., Chengdu, China	Analytical grade	N/A; decomposes before boiling
2,2′-Azobis[2-(2-imidazolin-2-yl) propane] dihydrochloride	Chengdu Kelong Chemical Co., Ltd., Chengdu, China	Analytical grade	N/A; undergoes thermal azo decomposition
tert-butyl hydroperoxide, 70 wt% aqueous solution	Chengdu Kelong Chemical Co., Ltd., Chengdu, China	Analytical grade	N/A; thermally unstable and decomposes on heating

Note: Boiling-point values refer to a pressure of 101.3 kPa. N/A indicates that a conventional normal boiling point is not applicable because the substance is a nonvolatile ionic solid or undergoes decomposition, polymerization, or another chemical transformation before boiling. Thermal-property information was obtained from the corresponding suppliers’ safety data sheets.

**Table 3:** Instruments and devices used.

Instrument or Device	Model	Manufacturer	Location
Ultrapure-water purification system	Arium Mini	Sartorius Lab Instruments GmbH & Co. KG	Göttingen, Germany
Overhead mechanical stirrer	1600	Qingdao ChuangMeng Instrument Co., Ltd.	Qingdao, China
NMR spectrometer	AVANCE NEO 600 MHz	Bruker BioSpin GmbH	Rheinstetten, Germany
Multi-angle light-scattering detector	DAWN HELEOS II	Wyatt Technology Corp.	Santa Barbara, CA, USA
SEC column	G6000PWXL	Tosoh Bioscience Shanghai Co., Ltd.	Shanghai, China
Differential refractometer	Optilab T-rEX	Wyatt Technology Corp.	Santa Barbara, CA, USA
Mechanical stirrer for SEC sample preparation	WiseStir HS-30E	Daihan Scientific Co.	Wonju, Repulic of Korea
Rheometer	HAAKE MARS 60	Thermo Fisher Scientific	Karlsruhe, Germany
3D laser scanning microscope	VK-X1000	KEYENCE Corporation	Osaka, Japan
Pipe-flow apparatus	Laboratory-built	Developed in-house	Chengdu, China

## Data Availability

The data presented in this study are available from the corresponding author upon reasonable request.

## Abbreviations

AM	acrylamide
AMPS	2-acrylamido-2-methylpropanesulfonic acid
DR	drag-reduction rate
LVR	linear viscoelastic region
NMR	nuclear magnetic resonance
PEEK	polyether ether ketone
RDR	post-shear drag-reduction retention
SD	standard deviation
SEC–MALS	size-exclusion chromatography coupled with multi-angle light scattering
